# Annual Commencement of Baltimore College of Dental Surgery

**Published:** 1842-03

**Authors:** 


*Baltimore College of Dental Surgery.*—The second annual public Commencement of the Baltimore College of Dental Surgery, was held in the Assembly Rooms, on the evening of Friday, February 18th, 1842.

The proceedings were commenced with prayer by the Rev. Job Guest. The Dean presented for graduation the following named gentlemen, upon whom severally the degree of Doctor of Dental Surgery was conferred by the President of the Faculty, Professor Hayden.

W. R. Scott, Raleigh, N. C.; Subject of Thesis, *Dental Caries.*
Wm. W. H. Thackston, Farmville, Va. " *Diseases of the Antrum.*
Jos. B. Savier, Norfolk, Va. " *Irregularities of the Teeth.*

The Dean then announced the following named gentlemen upon whom severally, the honorary degree of Doctor of Dental Surgery was conferred.

Eleazar Parmly, M. D. *New York;* Sir Samuel Cartwright, *London;* Levi S. Parmly, M. D. *Matanzas;* J. Starr Brewster, M. D. *Paris, France;* Leonard Koecker, M. D. *London;* Edward Maynard, M. D. *Washington, D. C;* —— Greenwood, *Boston;* J. F. Flagg, M. D. *Boston;* Daniel Harrington, *Philadelphia;* Edward Hudson, *Philadelphia;* Isaac I. Greenwood, M. D. *New York;* James S. Gunnell, M. D. *Washington, D. C.* J. Smith Dodge, *New York;* Ludolph Parmly, *Mobile;* A. B. Hayden, *Savannah, Geo*; Solyman Brown, M. D. *New York;* Jahial Parmly, *New York;* C. Hayden, *Washington, D. C.*

The Valedictory Address was then read by Professor W. R. Handy, and the services were closed by a benediction, pronounced by Rev. John N. M'Jilton. Thos. E. Bond, Jr. *Dean.*

At the first annual commencement the degree of Doctor of Dental Surgery was conferred on R. Covington Mackall and Robert Arthur, both of Baltimore. T. E. B. Jr.

